# Child Protection Service interference in childhood and the relation with mental health problems and delinquency in young adulthood: a latent class analysis study

**DOI:** 10.1186/s13034-017-0205-0

**Published:** 2017-12-21

**Authors:** Laura van Duin, Floor Bevaart, Carmen H. Paalman, Marie-Jolette A. Luijks, Josjan Zijlmans, Reshmi Marhe, Arjan A. J. Blokland, Theo A. H. Doreleijers, Arne Popma

**Affiliations:** 1 Department of Child and Adolescent Psychiatry, VU University Medical Center, Meibergdreef 5, 1105 AZ Amsterdam, The Netherlands; 2 Leiden Law School, Leiden University, Institute of Criminal Law and Criminology, PO Box 9520, 2300 RA Leiden, The Netherlands

**Keywords:** Child Protection Service, Latent classes, Multi-problem, Young adults, Delinquency

## Abstract

**Background:**

Most multi-problem young adults (18–27 years old) have been exposed to childhood maltreatment and/or have been involved in juvenile delinquency and, therefore, could have had Child Protection Service (CPS) interference during childhood. The extent to which their childhood problems persist and evolve into young adulthood may differ substantially among cases. This might indicate heterogeneous profiles of CPS risk factors. These profiles may identify combinations of closely interrelated childhood problems which may warrant specific approaches for problem recognition and intervention in clinical practice. The aim of this study was to retrospectively identify distinct statistical classes based on CPS data of multi-problem young adults in The Netherlands and to explore whether these classes were related to current psychological dysfunctioning and delinquent behaviour.

**Methods:**

Age at first CPS interference, numbers and types of investigations, age at first offence, mention of child maltreatment, and family supervision order measures (Dutch: ondertoezichtstelling; OTS) were extracted from the CPS records of 390 multi-problem young adult males aged 18–27 (mean age 21.7). A latent class analysis (LCA) was conducted and one-way analyses of variance and post-hoc t-tests examined whether LCA class membership was related to current self-reported psychological dysfunctioning and delinquent behaviour.

**Results:**

Four latent classes were identified: (1) *late CPS/penal investigation group* (44.9%), (2) *early CPS/multiple investigation group* (30.8%), (3) *late CPS interference without investigation group* (14.6%), and (4) *early CPS/family investigation group* (9.7%). The early CPS/family investigation group reported the highest mean anxiousness/depression and substance use scores in young adulthood. No differences were found between class membership and current delinquent behaviour.

**Conclusions:**

This study extends the concept that distinct pathways are present in multi-problem young adults who underwent CPS interference in their youth. Insight into the distinct combinations of CPS risk factors in the identified subgroups may guide interventions to tailor their treatment to the specific needs of these children. Specifically, treatment of internalizing problems in children with an early onset of severe family problems and for which CPS interference is carried out should receive priority from both policy makers and clinical practice.

## Background

Childhood onset of delinquent behaviour and severe family problems, including child maltreatment and neglect, are associated with a variety of adverse outcomes in young adulthood [1–6]. These childhood problems are important risk factors for later delinquent behaviour and hamper psychological functioning [1, 3, 4, 7–17]. So far, childhood risk factors of adulthood problems have been studied either within delinquent populations [1–3, 9, 13, 18–21] or in populations of young adults who experienced maltreatment and out-of-home placements in their childhood [3, 22]. These studies focused predominantly on the severity, age of onset and persistence of delinquent behaviour and on maltreatment and family interferences by, for example, the Child Protection Services (CPS; Dutch: *Raad voor de Kinderbescherming*). However, such childhood problems are closely interrelated and the presence of multiple problems in childhood drastically increases the probability of adverse adult outcomes [19, 23, 24]. Therefore, studies should focus on combinations of risk factors in young children [13, 25, 26], instead of focusing on single risk factors, and assess to what extent these combinations can predict outcomes later in life. In this way, it may be possible to distinguish among youth risk profiles which may help tailor primary, secondary and tertiary prevention strategies. The present study tackled these issues by retrospectively studying combined risk factors and long-term outcomes of both childhood judicial and civil CPS interferences in multi-problem young adults.

Young adulthood is considered a distinct developmental stage comprising major psychological [27–29], social [27] and neurobiological [30] changes that are critical for a healthy transition towards adulthood [31–33]. In most cases, young adults (aged 18–27) who experienced severe psychological, family and judicial problems since childhood encounter difficulties during this transition in becoming self-sufficient adults [32–35]. Previous studies have provided evidence that these vulnerable young adults are at high risk of an accumulation of several problems such as unemployment, psychological problems, early parenthood, and court involvement [34, 36–38]. Furthermore, a majority of these young adults suffer from substance use disorder [39, 40], and lack social support [33, 34]. This group with multiple and intertwined problems has been called multi-problem young adults, and is increasingly recognized as warranting specific scientific attention in order to inform and help improve professional support [33, 41]. An important aspect in this respect is to understand the development of the childhood problems that culminate in these multi-problem young adults.

In general, childhood problems as risk factors of later delinquent behaviour and mental health problems are widely studied. These risk factors are often distinguished on the individual and family level [2, 9, 12, 13]. Individual risk factors as intellectual disability, disruptive behaviour, psychological problems and an early onset of substance use are related to the development of antisocial behaviour [2, 42–44] later in life, and to mental health problems in adulthood as well [45]. Other risk factors in this respect are low school achievement and truancy [46, 47]. Important risk factors on the family level are inadequate parenting, low social economic status, maltreatment and neglect, mental health problems and substance abuse of parents [12]. All these factors may have contributed in their own unique way to the various problems of young adults.

Many multi-problem young adults have demonstrated delinquent behaviour and severe family problems during childhood [1, 22, 48–50] and, therefore, are likely to have underwent CPS interference during their youth. In The Netherlands, there are two main reasons for a child to receive a CPS investigation: to request a civil or a penal measure. It is not uncommon for children to receive multiple CPS interferences during their lives [3]. Therefore, the characteristics of CPS interference differ among children [21, 51–53]. Multi-problem young adults are likely to have experienced several judicial, school and family problems simultaneously [19, 23, 24], for which the timing, the number and the intensity of CPS investigations may vary [3]. CPS characteristics can be seen as static risk factors [54] for deviant development since children who underwent CPS interference have an elevated risk of developing delinquent behaviour and mental health problems in young adulthood [1, 3, 8, 21, 48, 55, 56]. The annual arrest rate for young adults who as children had been referred to CPS is more than four times higher than the national rate for 18- to 24-year olds [57] and 50% of this young adult population have experienced mental health problems [57].

Whereas all children who were exposed to severe family problems and/or who were involved in juvenile delinquency have an elevated risk of adult problem behaviour [1, 6, 15, 50, 58–61], the extent to which these problems persist and evolve into young adulthood differs substantially [7, 61, 62]. This might indicate heterogeneous profiles of the concurrent childhood problems. Several studies investigated and aimed to reduce the heterogeneity of problems within comparable populations of high-risk youths by exploring profiles [9, 13]. A study by Haapasalo found two groups of young adult offenders with CPS interventions: an early onset multiple intervention group and a late onset group who had fewer interventions [3]. A study by Dembo et al. [9] in high-risk youths reported two classes based on self-report data; one with a low prevalence and the other with a high prevalence of problems in family and peer relations, psychological functioning and education [9]. Furthermore, Geluk et al. [13] distinguished three profiles in childhood arrestees, differing in the extent of problems in peer relations, psychological functioning and authority conflicts. So, exploring profiles proved useful in ordering these childhood problems into several homogenous classes concerning the onset, the prevalence and the extent of the problems. However, these studies did not explore specifically if and how these childhood classes may contribute to a deviant development into (young) adulthood.

Although CPS does not provide treatment, CPS interference is directly related to extensive contact with judicial, mental health and social services [48, 63] and CPS may refer their clients to appropriate care, if necessary. However, many (young) adults with a childhood history of CPS interference still experience serious problems, even after repeated intervention [3, 48, 49, 64, 65]. As such, it seems that the effectiveness of current secondary prevention and intervention practices during childhood is limited in this population. Therefore, retrospectively identifying classes of interrelated static risk factors of CPS interference within a relatively unstudied population of multi-problem young adults may prove useful for more effective problem recognition and screening purposes in childhood [26, 54]. Finally, relating these childhood classes to delinquency and mental health problems in young adulthood may give useful indications for the prevention of the escalation of these problems to clinical practice [48, 49].

The present study aims to explore whether groups of CPS characteristics in childhood can be identified within a sample of multi-problem young adults. Furthermore, the associations between class membership and both self-reported delinquency and psychological functioning in young adulthood are investigated. Based on the literature, we expect multi-problem young adults to have a significant prevalence of CPS interference. Within this group we expect to find distinct latent classes differing in the onset, number and intensity of judicial and civil interferences [3] and in the extent of family problems [7, 9]. Lastly, it is hypothesized that classes of CPS interference in youths relate differently to current psychological dysfunctioning and current severity of delinquent behaviour in multi-problem young adults [1, 65, 66].

## Methods

### Study sample

In 2014–2016 a total of 596 multi-problem young adults were recruited in Rotterdam, The Netherlands. All participants were male, between 18 and 27 years old (mean age 21.7), and had sufficient knowledge of the Dutch language to understand the study procedure and the questionnaires. This study was part of a larger study in which participants were recruited from two sites. The first site was a municipal agency (Dutch: *Jongerenloket*) where young adults between the ages of 18 and 27 can apply for social welfare. Every year over 4000 intakes are carried out by so-called youth coaches [67]. During this intake, the level of self-sufficiency of the young adult is assessed on eleven life domains with the validated Self-Sufficiency Matrix—Dutch version (SSM-D) [68–70], based on the American version of the SSM [71], on a five-point scale with scores ranging from 1 (acute problems) to 5 (completely self-sufficient). Participants were eligible when they adhered to the following definition: (a) a score of 1 or 2 on the domains Income and Daytime Activities, (b) a maximum score of 3 on at least one of the following domains: Addiction, Mental health, Social network, Justice and (c) a minimum score of 3 on the domain Physical health [72]. Eligible young adults were asked to cooperate voluntarily. As a part of a larger study, *N* = 436 participants were recruited in this way [72]. The second site was multimodal day treatment program *New Opportunities* (Dutch: *De Nieuwe Kans*; DNK). Multi-problem young adults also signed up to DNK themselves or were referred to DNK directly by youth care, probation services, mental health services or social organizations. Therefore, additional participants were recruited directly from DNK (*N* = 160). From the total study sample (*N* = 596), 99.3% (*N* = 592) gave informed consent to conduct the register and record research. Of the *N* = 592, 65.9% (*N* = 390) was matched to a record in the CPS system.

### Procedure

The study was performed by the VU University Medical Center Department of Child and Adolescent Psychiatry and approved by the Medical Ethics Review Committee of VU University Medical Center.^1^ Participants gave informed consent before voluntary participation after a member of the research team had provided oral information accompanied by written information. After informed consent, trained (junior) researchers administered questionnaires.

Interference with CPS was checked in the CPS system *Kinderbescherming Bedrijfs Processen Systeem* (KBPS) using first names, surname and date of birth of the participants. This resulted in a match of 65.9% (N = 390) of the total sample (N = 592); 34.1% (N = 202) did not match to a record in the system. For a part of the latter group it is uncertain whether they truly never had CPS contact or whether their record has been destroyed, since CPS is legally required to destroy records of clients that reach age 24. This applies to N = 98 of the N = 202 that did not match to a record in the system. For the other N = 104 (51.5% of N = 202), it was certain that they did not have CPS interference, since they were younger than 24 years old. The CPS files consist of all documents received and sent by the CPS concerning the child and a selection of judicial and police report data [73]. Data were extracted from April 2015 to August 2016 by trained (junior) researchers. To test the inter-rater reliability, 19 randomly selected files were scored by two independent raters, showing a substantial inter-rater reliability (κ = 0.72) [74, 75].

### Context

The register and record research was conducted at CPS and the data were extracted between April 2015 and August 2016. CPS monitors children between 0 and 18 years old when there are serious concerns regarding their home situation and upbringing. In families with severe parenting problems a child welfare investigator can perform a civil protection investigation of the home environment of the child, at the request of CPS. At the request of the court, CPS mediates when parents break up and disagree about arrangements concerning their children. Moreover, CPS can initiate a judicial or truancy investigation for youth suspected of an offence or truancy. The investigation report with recommendations on (mandatory) service use or a suitable penalization is delivered to the court [73].

### Measurements

#### Socio-demographic characteristics

Socio-demographic characteristics were assessed with a structured self-report questionnaire. *Ethnicity* was based on the country of birth of the respondent and at least one of his parents. A respondent was classified as non-Dutch if he or one of his parents was not born in The Netherlands [76]. Ethnicity was recoded into a dichotomous variable (Dutch ethnicity vs. other ethnicity). *Educational level* was classified into three levels: maximum primary education, achievement of junior secondary education and senior secondary education attainment. *Family problems in youth* were assessed with the single item ‘Did you suffer from problems that existed in the family you grew up with? (Yes/No)’. *Police contact of family members in youth* was assessed with the single item ‘Did family members you grew up with have police contact? (Yes/No)’. *Prior service use* was assessed with the single item ‘Did you previously use services? (Yes/No)’. *Frequency of service use* was assessed with the single item ‘Which services did you have contact with?’ (e.g., youth care, probation services, child protection services). This was recoded into a frequency score defined as the number of self-reported services.

#### CPS variables

Several variables were obtained from the CPS records. All variables were divided into categories to perform the latent class analysis (LCA), as it is a condition for this analysis to use categorical variables. The variables Age of first CPS report, Type of investigation, Number of investigations, Child maltreatment, Age of onset of delinquent behaviour and Family supervision order were used as indicators to execute the LCA. *Age of first CPS report* in which date of the first CPS investigation was recoded into four categories: no report, below age 13, 13 or 14 years old, age 15 up to 18. The CPS records provided information on three types of investigations: offence investigation, protection investigation and truancy investigation. *Type of investigation* was recoded into a variable that contained five categories: no investigation, protection investigation, offence investigation, truancy investigation, several types of investigations. *Number of CPS investigations* was recoded into three categories: no investigation, one or two investigations, at least three investigations. *Child maltreatment* was extracted from the record when a professional ascertained child maltreatment (Yes/No). *Domestic violence* was observed and registered by a professional (Yes/No). The verdict of the court to impose a *family supervision order* was included in the record (Yes/No). *Out*-*of*-*home placement* was also included in the record in the verdict of the court (Yes/No). *Age of onset of delinquent behaviour*: the date of the first offence was registered based on the police report. Using this date combined with the date of birth, the age of first offence was computed. This variable was recoded into four categories: no offence, first offence below age thirteen, first offence between 13 and 14 years of age, and first offence at age 15 or older.

#### Current psychological functioning

The Dutch version of the Adult Self Report (ASR) [77] was assessed orally and filled out by the researcher to obtain current psychological functioning. ASR part VIII consists of 123 items on internalizing and externalizing problems during the previous 6 months. The reliability of the questionnaire is good, with a Cronbach’s α of 0.83. In this study the ASR total problem score and the scores of nine subscales were used as outcome measures. The subscales are: anxious/depressed, withdrawn, somatic complaints (internalizing problems); intrusive, rule-breaking and aggressive behaviour (externalizing problems); thought problems, attention problems and substance use. The prevalence of serious dysfunctioning on all subscales is presented in Table 1. The mean scale scores per class as outcome measure are based on percentile scores [78] (Table 5).

**Table 1:** Descriptive characteristics in percentages (*N* = 390*)*

Socio-demographic characteristics	
Mean age	21.7 years old
Born in The Netherlands	
Yes	76.6
Dutch ethnicity	
Yes	12.6
Educational level	
Primary	36.5
Junior secondary	44.7
Senior secondary	17.5
Other	1.3
Family characteristics	
Family problems in youth	
Yes	63.2
Police contact of family members in youth	
Yes	19.0
Service use	
Service use	
Yes	83.3
Frequency of service use	
None	16.2
Once	28.0
2 or 3	36.5
4 or more	19.3

	Prevalence serious dysfunctioning (%)^a^
Psychological functioning previous 6 months (ASR)	
Total problems	29.8
Anxious/depressed	30.8
Withdrawn	51.2
Somatic complaints	29.3
Intrusive	7.7
Rule-breaking behaviour	44.7
Aggressive behaviour	28.0
Attention problems	30.6
Thought problems	34.2
Substance use	53.0
Delinquent behaviour from onset till young adulthood (SRD)	
Committed at least one offence	
Yes	93.3
Destruction/public order offence	
Yes	62.6
Property offence	
Yes	85.9
Aggression/violent offence	
Yes	73.1
Drug offence	
Yes	59.2
Delinquent behaviour previous 6 months (SRD) (N = 179)^b^	
Committed at least one offence	
Yes	63.0
Destruction/public order offence	
Yes	10.8
Property offence	
Yes	27.1
Aggression/violent offence	
Yes	21.6
Drug offence	
Yes	21.0

^a^Prevalence of serious dysfunctioning is based on percentile scores in the borderline (between the 84th and 90th percentiles) and clinical range (above the 90th percentile) [78]

^b^Self-reported delinquency in the previous 6 months has been added during the study and measured in 179 participants

#### Delinquent behaviour

The frequency and seriousness of delinquent behaviour were investigated orally and filled out by a researcher using the Dutch version [79] of the Self-report Delinquency Scale (SRD) [80]. This questionnaire has 29 items (including two items of violation: fare dodging and lighting fireworks when prohibited) and the internal consistency of the total score is excellent with Cronbach’s *α* = 0.85 [79, 81]. The questionnaire explored the frequency of offences committed both during the respondent’s lifetime and in the previous 6 months. In addition, the items were also divided into four different offence categories: destruction/public order offences (5 items, Cronbach’s *α* = 0.64), property offences (11 items, Cronbach’s *α* = 0.79), aggression/violent offences (8 items, Cronbach’s *α* = 0.7) and drug offences (3 items, Cronbach’s *α* = 0.72) [79]. The frequencies per offence category were recoded into dichotomous variables (Yes/No), due to the skewed distribution of the data. Lifetime and previous 6 months’ prevalence are presented in Table 1. Mean scores based on the frequencies of offences in the previous 6 months were used as outcome measure (see Table 5). The 27 items (excluding two items of violation) add up to one total delinquency score reflecting the multiplication of the seriousness of the offences and their frequency. The seriousness is divided into minor and serious offences based on applicable legal penalties; minor offences have a maximum custodial sentence of 48 months (score 1) and serious offences have a minimum custodial sentence of 48 months (score 2) [79, 80].

### Data analysis

In order to detect classes of childhood correlates Latent Class Analysis (LCA) was performed. LCA is a useful method for analysing the relationships among observed variables, when each observed variable is categorical, in a heterogeneous population assumed to be comprised of a set of latent classes [82]. LCA was performed with the program Statistical Analysis System (SAS) version 9.3. The six CPS childhood indicators mentioned above were entered into the LCA. Analyses were conducted using PROC LCA 1.2.6 for SAS 9.3 [83]. Good qualification quality was established taking into account the Bayesian information criterion (BIC), entropy and Akaike information criterion (AIC) [82]. The entropy value ranges between 0 and 1; a value approaching 1 indicates a clear description of the classes [84]. Subsequently, item response probability scores on all indicators were used to interpret the classes. Lastly, to explore differences among classes derived from the LCA on current psychological functioning and delinquent behaviour, One-Way Analyses of Variance and Post Hoc t-tests with Bonferroni correction were performed with Statistical Packages for the Social Sciences, version 22 for Windows [85].

## Results

Table 1 shows the self-reported socio-demographic and family characteristics, service use, current psychological functioning and delinquent behaviour of multi-problem young adults with CPS interference in youth. It shows that many young adults had problems in youth; 63.2% had problems in their family, 83.3% reported prior service use and 93.3% committed an offence. During the previous 6 months, 53.0% had serious substance use problems and 63.0% committed an offence.

### Childhood correlates of the CPS records

Table 2 shows the descriptive results of the childhood CPS correlates in percentages. After referral to CPS, 84.9% of participants were investigated. In 21.0% of the participants the first CPS investigation was below the age of thirteen and 39.0% had their first investigation at age fifteen or older. Almost half of the group (43.9%) had one or two CPS investigations and 41.5% had at least three CPS investigations. Judicial investigations were conducted in 75.0% of the group and protection investigations in 40.0% of participants. Multiple types of investigations were conducted in 32.6% of participants of which 50.0% first had a protection investigation and 40.0% first had a judicial investigation. Truancy investigations rarely occurred separately (1.8%). Child maltreatment was registered in 29.5% of the CPS reports and the CPS records reported domestic violence in 16.4% of the cases. Protection measures taken by the juvenile court were investigated as well; 33.6% of participants underwent a family supervision order and 22.1% an out-of-home placement. In 88.5% of the CPS records childhood delinquency was registered and 23.3% committed their first offence below age 13.

**Table 2:** Frequencies of childhood correlates CPS records (*N* = 390)

	%
Age of the first CPS report	
No report	15.1
First report below age 13	21.0
First report age 13 or 14	24.9
First report age 15 or older	39.0
Number of CPS investigations	
None	14.6
1 or 2	43.9
3 or more	41.5
Type of CPS investigation	
No investigation	14.9
Protection investigation	8.0
Judicial investigation	42.7
Truancy investigation	1.8
Multiple types of investigations	32.6
Registered child maltreatment	
Yes	29.5
Domestic violence	
Yes	16.4
Family supervision order	
Yes	33.6
Out-of-home placement	
Yes	22.1
Age at onset of delinquent behaviour	
No offence	10.5
Below age 13	23.3
Age 13 or 14	33.6
Age 15 or older	32.6

### Identification of childhood correlate classes (Latent Class Analysis)

The first step conducted for the LCA involved identifying the number of latent classes that best fit the data on six childhood indicators. Table 3 presents the fit indices after carrying out several class models. Based on the entropy (0.95) and the BIC value (692.03), the four-class models fitted best. The five-class model, however, had the lowest value of the AIC (417.74). Models distinguishing six or more classes all performed worse on all indicators. Based on these findings and the interpretability of the resulting latent class model, we decided that the four-class model had the best fit for these data.

**Table 3:** Model fit sizes of latent class analysis of childhood correlates (*N* = 390)

Model	Entropy	AIC	BIC	Df
2	1.00	1009.57	1124.58	930
3	0.93	597.93	772.44	915
4	0.95	458.02	692.03	900
5	0.91	417.74	711.24	885

*AIC* Akaike information criteria, *BIC* Bayesian information criteria; *Df* degrees of freedom

In order to interpret the latent classes, item response probabilities of the indicators were examined for each latent class. Table 4 presents the item-response probabilities and the proportions of the classes.

**Table 4:** Item response probabilities LCA (*N* = 390)

Class	1 (*N* = 175)	2 (*N* = 120)	3 (*N* = 57)	4 (*N* = 38)
Class size proportions	44.9%	30.8%	14.6%	9.7%
Family supervision order				
Yes	0.02	*0.84*	0.02	*0.70*
No	*0.98*	0.16	*0.98*	0.30
Registered child maltreatment				
Yes	0.14	*0.57*	0.02	*0.59*
No	*0.86*	0.43	*0.98*	0.41
Age at onset of delinquent behaviour				
No offence	0.00	0.00	0.31	*0.62*
Below age 13	0.20	0.42	0.05	0.10
Age 13 or 14	0.41	0.37	0.18	0.11
Age 15 or older	0.39	0.21	0.46	0.18
Age of the first CPS report				
No report	0.01	0.01	*0.997*	0.00
First report below age 13	0.04	0.44	0.00	*0.60*
First report age 13 or 14	0.29	0.34	0.00	0.15
First report age 15 or older	*0.67*	0.21	0.00	0.25
Number of CPS investigations				
None	0.00	0.00	*0.997*	0.00
1 or 2	*0.68*	0.13	0.00	*0.94*
3 or more	0.32	*0.87*	0.00	0.06
Type of CPS investigation				
No investigation	0.00	0.00	*0.997*	0.03
Protection investigation	0.00	0.00	0.00	*0.85*
Judicial investigation	*0.89*	0.04	0.00	0.12
Truancy investigation	0.04	0.00	0.00	0.00
Multiple types of investigations	0.07	*0.95*	0.00	0.00

Current psychological functioning and delinquent behaviour per group

**Fig. 1:**
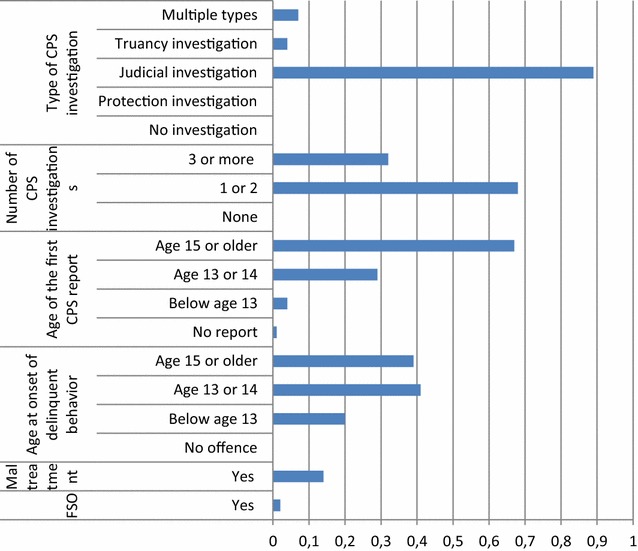
1-Late CPS/penal investigation group

The first class, labelled as the *late CPS/penal investigation group* (44.9%) (Fig. 1), did not experience maltreatment or a family supervision order in childhood. They all committed at least one offence^2^ and their first offence was at age 13 or 14. Their first judicial CPS report was executed at age fifteen or older (late CPS interference) and they had a maximum of two, solely judicial, reports.

**Fig. 2:**
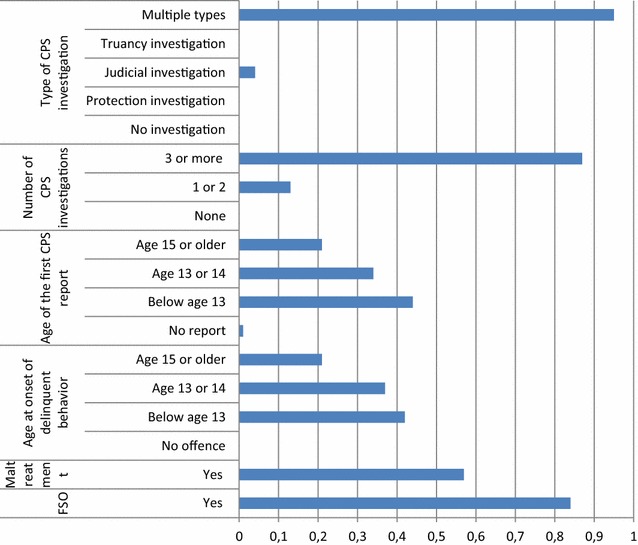
2-Early CPS/multiple investigation group

A majority of the second class, labelled as the *early CPS/multiple investigation group* (30.8%) (Fig. 2), experienced maltreatment in childhood which often resulted in at least one family supervision order pronounced by the court. They had their first report at a young age, below age 13 (early CPS interference) and had three or more CPS investigations, due to various causes (judicial and/or family and/or truancy investigations), since they often committed their first offence below age thirteen.

**Fig. 3:**
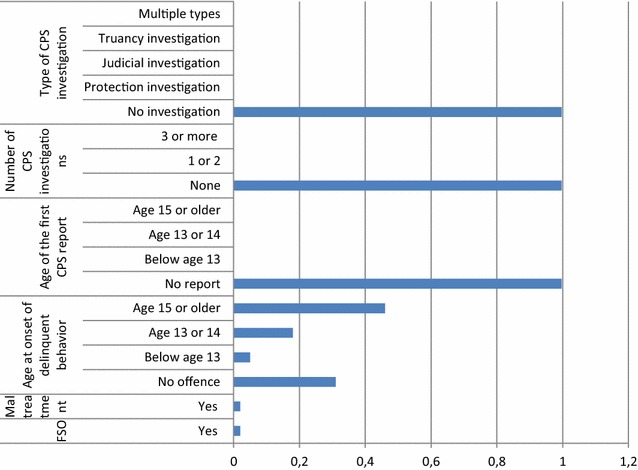
3-Late CPS interference without investigation group

The third class, labelled as the *late CPS interference without investigation group* (14.6%) (Fig. 3), did not experience any severe family problems such as maltreatment or family supervision orders. If they committed an offence, it was at age 15 or older (late CPS interference). CPS decided mostly not to investigate the child and they often did not have any reports in their record.

**Fig. 4:**
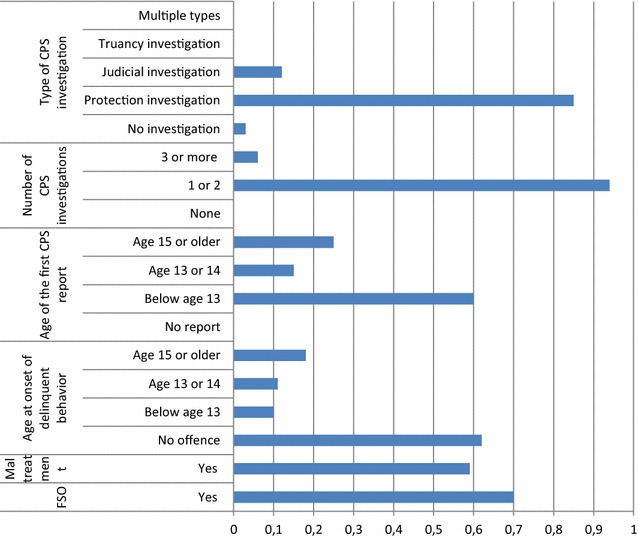
4-Early CPS/family investigation group

The fourth class, labelled as the *early CPS/family investigation group* (9.7%) (Fig. 4), had early CPS interference below age thirteen (early CPS interference), due to severe family problems such as maltreatment which resulted mostly in at least one family supervision order. CPS decided to investigate their situations once or twice, which were specifically protection investigations. Participants in this group were not likely to commit any offence.

Table 5 presents results of the ANOVA and post hoc comparisons between LCA class membership on current psychological functioning. There was a significant difference among classes on anxious/depressive problems (*p* = 0.035), a borderline significant difference on intrusive problems (*p* = 0.056) and a significant difference on substance use (*p* = 0.029). The post hoc test showed that participants of the early CPS/family investigation group reported significantly more anxious/depressive problems than participants of the early CPS/multiple investigation group (*p* = 0.022). Moreover, the early CPS/family investigation group reported more substance abuse than the late CPS interference without investigation group (borderline significant; *p* = 0.056).

**Table 5:** Results of ANOVA comparisons among classes on current self-reported psychological functioning and delinquent behaviour (*N* = 390)

Class	1 (*N* = 175)	2 (*N* = 120)	3 (*N* = 57)	4 (*N* = 38)	F	p
	M (SD)	M (SD)	M (SD)	M (SD)		
Psychological functioning^a^						
Total psychological problems	61.4 (26.0)	61.5 (25.8)	59.8 (28.2)	71.1 (22.8)	1.71	0.164
Anxious/depressed	69.2 (18)	66.3 (16)	69.4 (18)	75.8 (18)	2.88^b^	0.035**
Withdrawn	79.0 (17.2)	78.1 (16.8)	73.2 (18.7)	80.8 (16.6)	1.97	0.118
Somatic complaints	68.1 (16.4)	67.8 (16.1)	69.2 (17.4)	72.6 (16.7)	0.90	0.439
Intrusive	55.7 (1)	59.3 (1)	55.7 (1)	57.8 (2)	2.55^c^	0.056*
Rule-breaking behaviour	78.6 (16.8)	79.9 (16.8)	78.4 (16.2)	82.6 (17.6)	0.71	0.549
Aggressive behaviour	67.7 (16.1)	67.2 (15.5)	68.5 (17)	74.2 (16.9)	1.97	0.118
Attention problems	73.4 (14.3)	74 (14.5)	72.3 (14.5)	77.7 (14.7)	1.18	0.317
Thought problems	74.3 (17.5)	73.2 (16.3)	72.1 (17.3)	79.2 (16.5)	1.52	0.208
Substance use^d^	78.0 (18)	81 (19)	73.9 (19)	83.9 (18)	3.04^e^	0.029**

Class	**1** (*N* = 74)	**2** (*N* = 59)	**3** (*N* = 25)	**4** (*N* = 21)	F	p
	M (SD)	M (SD)	M (SD)	M (SD)		
Delinquency previous 6 months						
Total delinquency	3.5 (8.1)	7.1 (11.5)	6.0 (13.2)	2.2 (5.3)	2.1	0.101
Destruction/public order offence	0.09 (0.3)	0.14 (0.4)	0.00 (0)	0.19 (0.4)	1.89	0.133
Property offence	0.22 (0.4)	0.37 (0.5)	0.27 (0.5)	0.18 (0.4)	1.72	0.165
Aggression/violent offence	0.20 (0.4)	0.27 (0.4)	0.15 (0.4)	0.23 (0.4)	0.61	0.609
Drug offence	0.57 (0.5)	0.65 (0.5)	0.54 (0.5)	0.61 (0.5)	1.35	0.261

^a^Normal functioning (score < 84), borderline range (score 84-90), clinical range (above 90) [78]

^b^Significant difference between early CPS/family investigation group and early CPS/multiple investigation group

^c^Significant difference between early CPS/multiple investigation group and late CPS/penal investigation group

^d^Class 1; *N* = 174

^e^Significant difference between early CPS/family investigation group and late CPS interference without investigation group

* *p* < 0.10, ** *p* < 0.05, *** *p* < 0.01

No significant differences among LCA classes were found on self-reported current delinquent behaviour (Table 5).

## Discussion

The purpose of this study was twofold. The first aim was to retrospectively identify distinct classes in multi-problem young adults based on childhood CPS characteristics. This resulted in four latent classes: a late CPS/penal investigation group (44.9%), an early CPS/multiple investigation group (30.8%), a late CPS interference without investigation group (14.6%) and an early CPS/family investigation group (9.7%). The second aim was to explore whether these classes differed on current young adult psychological functioning and delinquent behaviour. The early CPS/family investigation group reported significantly more problematic anxiousness/depression problems than the other groups. Substance use differed significantly among groups, although post hoc tests only revealed borderline significant differences. No differences in current delinquent behaviour were reported among the classes.

In our sample of multi-problem young adults, 65.9% had one or more CPS interference(s) during their childhood versus 1% of the total population of Dutch children in 2016 [86]. Furthermore, 29.5% in the current sample underwent maltreatment versus 3% of Dutch youth that was in danger of any type of maltreatment in 2010 [87]. Thus, the prevalence of CPS interferences and severe family problems is, as expected, clearly higher in this population of multi-problem young adults than in the general population. One should note, however, that these percentages are not completely comparable, since the prevalence in the current study was not limited to 1 year. The high prevalence of CPS interference in multi-problem young adults matches their self-reported problems in childhood quite adequately: 83.3% reported service use in their youth and 63.2% reported family problems. As expected, multi-problem young adults also experience heterogeneous problems in their current functioning. This extends findings in other studies [88–90] that argue that different forms of problem behaviour (such as mental health problems, delinquency and substance use) with an onset in childhood are interrelated and may be seen as symptoms of a general disposition toward deviant behaviour through life, by some referred to as problem behaviour syndrome (PBS) [91]. How PBS is expressed may vary over time and across contexts. For children with PBS, the transition to adulthood typically occurs in the context of severe family problems and interference by multiple justice/care/and child welfare systems [41, 66]. Therefore, they may experience a differential pathway into adulthood in which more tailor-made specialized care is needed to support their adopting adult responsibilities such as independent living [41]. This way, they may be prevented from growing into multi-problem young adults. Our first findings underline the importance of gaining more insight into the childhood onset of the problem heterogeneity of multi-problem young adults in order to enhance effective tailor-made intervention.

The present study confirmed several distinct classes of risk factors for adult problem behaviour in addition to earlier studies [3, 9, 13]. Dembo et al. 9 and Geluk et al. 13 identified two and three classes, respectively, differing in the extent of problem behaviour; Haapasalo [3] reported two classes differing in age of onset and number of CPS interventions. A first distinction in the identified classes in the current study indeed occurred between early (below age 13) and late (from age 15) CPS involvement. The early CPS/multiple investigation group had the earliest onset of delinquent behaviour (below age 13). Several studies show that early onset delinquents are more at risk for problems in young adulthood, such as mental health problems, substance abuse, drug related and violent delinquent behaviour, than later onset delinquents [20, 61]. Furthermore, the early CPS/multiple investigation group underwent the most CPS investigations and is, therefore, also comparable to the early onset group in the Haapasalo study [3], in which the offenders demonstrated more problems during their youth and were in greater need of CPS interventions such as placement in foster care.

Regarding the long term outcomes of childhood CPS interference specifically, the early CPS/family investigation group reported the most anxious/depression problems and the most substance abuse in young adulthood. Maltreatment, family supervision and other severe family problems in childhood have repeatedly been shown to be robust risk factors for mental health problems in (young) adulthood [7, 16]. For example, according to Thornberry et al. [15], childhood maltreatment is indeed strongly related to later substance abuse and internalizing problems. Although the early CPS/family investigation was the smallest identified group (9.7%), they seem to have followed the most adverse developmental pathway into young adulthood. It is possible that CPS failed to provide appropriate interventions for this group, since the CPS involvement was not as intensive as for the early onset/multiple investigation group. Moreover, the early CPS/family group was the only group that did not engage in delinquent behaviour in childhood/adolescence. This may have caused them to stay unnoticed for a longer period of time. However, traumatic events in the child’s family environment may have already occurred long before the first CPS interference and are associated with an increased likelihood of adverse adult outcomes [7, 16]. Besides a broader focus on the problems of the child itself, children with solely civil CPS interference may benefit from more attention to treatment of the problems of the parents. Interventions could be aimed at strengthening their parenting capabilities and resources. Adopting such a ‘two-generation approach’ has shown promising results in preventing family and childhood problems from growing worse [92].

No significant differences among classes in current delinquent behaviour were found among groups. The late CPS/penal group was the largest group in our sample (44.9%); their first CPS investigation was at age 15 or older and the age of onset of their delinquent behaviour varied between ages 13 and 15. All multi-problem young adults showed a strong tendency for persisting in and/or developing criminal behaviour into adulthood, notwithstanding their distinct childhood histories. Moreover, since the group without CPS investigations also reported delinquent behaviour in adulthood, all forms of CPS interference (even marginal contact) should be considered risk factors for later antisocial behaviour. In addition, the late CPS/penal children proved to be a group without severe family problems, at least according to the CPS data. Steinberg [17] noted that adolescent onset offenders often manifest less severe patterns of family pathology and mental health problems than life course persistent offenders [61]. In our sample, both late onset CPS groups indeed reported fewer mental health problems in young adulthood than the early onset groups. A follow-up study should be conducted to explore whether these differences in problem behaviour among groups still persist into (middle) adulthood. Finally, since all groups persisted in their delinquent behaviour, children with CPS interference should be targeted as a high-risk population in need of specialized interventions aimed at reducing the criminogenic risk factors associated with recidivism.

## Limitations

Like any other study, this study has some limitations. First, the CPS record investigation in the current study was not performed using a validated instrument, because an applicable instrument was not available. However, CPS investigations are standardized and in order to optimize and monitor the quality of the data, inter-rater reliability was analysed and found to be substantial. Second, registered offence data, and in particular data on the first offence, is likely to be under reported, as a minority of juvenile delinquents is actually convicted [24]. Still, in this sample officially recorded and self-reported delinquency data are, while not exactly similar, quite comparable, both showing a high prevalence of delinquent behaviour. Third, in this study, self-report questionnaires were also used to investigate socio-demographic characteristics and psychological functioning. To achieve good reliability, a validated self-report psychological functioning questionnaire is used and anonymity and privacy of participants was emphasized before and during the assessment of questionnaires. Fourth, a majority of 87.4% of participants in this study have a non-Dutch ethnicity. In our case, non-Dutch ethnicity refers to an amalgam of cultural backgrounds, for example Surinamese, Antillean, Moroccan and Turkish. However, due to small sample sizes per ethnic subgroup, it was not possible to perform separate analyses. Fifth, generalizability of study results to an international context is not straightforward, because of different service system organizations. In Great-Britain and the United States of America, for example, child protection service and the judicial youth system are more separate systems than in The Netherlands [93, 94]. Scandinavian countries have more comparable systems to the Dutch system, although those systems are more based on prevention. For instance, in Sweden voluntary and involuntary services are not divided as in The Netherlands [95]. And lastly, LCA is an exploratory data-driven method and the findings per class represent probabilities on latent indicators.

## Conclusions

This study adds to the concept that even in a highly complex sample of multi-problem young adults who underwent CPS interference in their youth distinct developmental pathways, at least for mental health problems, can be distinguished. Although this exploratory study was not intended to produce definite ideas on how the underlying latent subgroups may experience differential treatment effects, our findings do suggest that members of the groups might benefit from interventions specifically tailored to their differing patterns of problems. The development of specific secondary and tertiary prevention programmes for children with an early onset of CPS interference and severe family problems should receive priority from both policy makers and clinical practice. In addition, evidence based interventions should be developed to prevent problem behaviour of all children that underwent CPS interference in their youth to prevent mental health problems and the persistence of delinquent behaviour into (young) adulthood.

## Abbreviations

AIC: akaike information criterion
ANOVA: analysis of variance
ASR: adult self report
BIC: Bayesian information criterion
CAU: care as usual
CPS: Child Protection Services
Df: degrees of freedom
DNK: New Opportunities (Dutch: *De Nieuwe Kans*)
KBPS: Kinderbescherming Bedrijfs Processen System (*CPS system*)
LCA: latent class analysis
M: mean
SAS: Statistical Analysis System
SD: standard deviation
SPSS: Statistical Packages for the Social Sciences
SRD: Self-Report Delinquency Scale
SSM-D: Self-Sufficiency Matrix-Dutch Version

## Footnotes

1 Registration number: 2013.422—NL46906.029.13.

2 Those who committed no offence in youth, have not (yet) experienced the onset of delinquency. Therefore, the category ‘no offence’ is mentioned in Table 4. For classes 1 and 2 this translates into all respondents in these classes having committed at least one offence.
